# Autoimmune Encephalitis as Treatment-Responsive Cause of Rapidly Progressive Dementia: A Multicenter Prospective Cohort Study

**DOI:** 10.1212/WNL.0000000000214933

**Published:** 2026-05-15

**Authors:** Robin W. van Steenhoven, Anna E.M. Bastiaansen, Jeroen Kerstens, Yvette S. Crijnen, Tessa Brand, Juliette Brenner, Tessa M. Bienfait, Juna M. de Vries, Yoav D. Piura, Nihal Satyadev, Marienke A.A.M. De Bruijn, Mariska M.P. Nagtzaam, Ece Erdag Turgeon, Suzanne C. Franken, Sharon Veenbergen, Rutger K. Balvers, Marc Langedijk, Robert M. Verdijk, Marcel M. Verbeek, Charlotte E. Teunissen, Yolande A.L. Pijnenburg, Frank Jan De Jong, Annemieke J.M. Rozemuller, Nina L. Fransen, Elise G.P. Dopper, Peter A.E. Sillevis Smitt, Gregory S. Day, Harro Seelaar, Maarten J. Titulaer

**Affiliations:** 1 Department of Neurology, Erasmus University Medical Center, Rotterdam, the Netherlands;; 2 Department of Neurology, Antwerp University Hospital, Belgium;; 3 Department of Neurology, Mayo Clinic in Florida, Jacksonville;; 4 Department of Neurology, Elisabeth-TweeSteden Ziekenhuis, Tilburg, the Netherlands;; 5 Department of Medical Immunology, Erasmus University Medical Center, Rotterdam, the Netherlands;; 6 Department of Neurosurgery, Erasmus University Medical Center, Rotterdam, the Netherlands;; 7 Department of Neurology, Treant Hospital, Emmen, the Netherlands;; 8 Department of Neuropathology, Erasmus University Medical Center, Rotterdam, the Netherlands;; 9 Department of Neurology and Human Genetics, Donders Institute for Brain Cognition and Behavior, Radboud University Medical Center, Nijmegen, the Netherlands;; 10 Neurochemistry Laboratory, Department of Chemistry, Amsterdam University Medical Center, the Netherlands;; 11 Department of Neurology, Amsterdam University Medical Center (VUMC), the Netherlands; and; 12 Department of Neuropathology, Amsterdam University Medical Center, the Netherlands.

## Abstract

**Background and Objectives:**

Early recognition of patients with rapidly progressive dementia (RPD) attributed to autoimmune encephalitis (AE) is important because rapid initiation of immunotherapy improves outcomes. However, ancillary testing is often time-consuming because of the broad differential diagnosis, highlighting the need for accurate patient selection based on early clinical features. We aim to determine the frequency of AE subtypes in patients with RPD (AE-RPD) and characterize presenting subphenotypes (i.e., symptoms in addition to dementia) compared with other diagnoses.

**Methods:**

This prospective multicenter observational cohort study was conducted from December 2019 to December 2024 across centers in the Netherlands and included adult patients with RPD, defined as dementia beginning within 1 year of symptom onset. Clinical features and ancillary testing data were collected and reviewed by 3 neurologists. Serum and CSF were evaluated for neuronal/glial autoantibodies.

**Results:**

A total of 147 patients were included (46% female; median age 67 years, range 36–86). AE-RPD was the largest diagnostic category (58/147; 39%) and most common treatment-responsive cause of RPD (58/95; 61%). Neurodegenerative diseases (20/147; 14%) and Creutzfeldt-Jakob disease (CJD, 17/147; 12%) accounted for the largest nonresponsive causes of RPD. RPD accompanied by seizures at first presentation was more frequent in AE-RPD compared with other diagnoses (20/58; 34% vs 9/89; 10%; *p* < 0.001), with anti-LG1 (11/20; 55%) encephalitis representing the most frequent underlying subtype. Seizures manifested as subtle focal events in 9 of 20 patients with AE-RPD (42%), complicating early clinical identification. Movement disorders were observed at presentation in similar proportions of patients with AE-RPD (17%) and CJD (35%; *p* = 0.11) but were more likely to emerge within 3 months of symptom onset in patients with RPD-AE (80% vs 25%; *p* = 0.004). In patients with AE-RPD without seizures, autoimmune glial fibrillary acidic protein (GFAP) astrocytopathy was the most common subtype (8/31; 26%), mostly presenting with prominent psychotic features or movement disorders (both 4/8; 50%).

**Discussion:**

In this study, AE was the most common treatment-responsive cause of RPD. Anti–leucine-rich, glioma-inactivated 1 encephalitis and autoimmune GFAP astrocytopathy were the predominant subtypes in AE-RPD.

## Introduction

Rapidly progressive dementia (RPD) is generally defined as the development of dementia within 1 or 2 years of symptom onset.^1-3^ The syndrome represents a significant challenge to clinicians because the differential diagnosis of RPD is broad and includes various treatment-responsive diseases, as opposed to dementia with a slowly progressive course.^1,2,4-7^ Consequently, RPD often necessitates an extensive and time-consuming diagnostic evaluation, while the time to act is limited because treatment effects are often most beneficial in early disease stages.^8^ Consequently, organization and prioritization of ancillary testing is essential to support early recognition of patients with potentially treatment-responsive causes of RPD and to prevent harmful diagnostic delays.^1,8,9^ In addition, early diagnostic certainty of nontreatable diseases, most notably Creutzfeldt-Jakob disease (CJD), is crucial for patients and caregivers because it enables effective counseling during the early stages of the disease and enables enrollment in clinical trials of putative disease-modifying therapies for prion disease.^1,8^

A major improvement in the diagnostic evaluation of RPD in the past decades is the discovery of autoimmune encephalitis (AE) and various associated disease-specific autoantibodies.^10^ Previous premortem and postmortem studies identified AE as important treatment-responsive mimic of CJD and neurodegenerative dementia.^1,11-14^ Early recognition of AE is important because early treatment is associated with improved short-term and long-term outcomes.^15-17^ More specifically, neuronal autoantibody status provides relevant information regarding tumor association, preferred treatment strategies, relapse risk, and longer term outcomes.^18^ In addition, identification of AE at a later onset age has been shown to be more complicated because of absence of direct evidence of inflammation, such as mesiotemporal hyperintensities on brain MRI or pleocytosis in CSF, and a broader differential diagnosis including various neurodegenerative diseases and comorbidities.^19,20^

Various RPD cohorts have been published since the first descriptions of AE, with the proportion of cases attributed to AE (AE-RPD) varying from approximately 5% to 30%.^1,6,7,9,21^ However, extensive descriptions on the early clinical presentation of AE and different subtypes are scarce. Filling this gap is important because proper targeted ancillary testing and autoantibody studies facilitate early diagnosis and treatment. We sought to determine the frequency of AE in a large prospective cohort of RPD. In addition, we comprehensively characterized the early clinical presentation of different AE subtypes in comparison with other causes of RPD to improve early recognition of patients with AE-RPD.

## Methods

### Study Design and Participants

The Erasmus University Medical Center (EMC) is the Dutch national referral center for neuroinflammation and accredited European Reference Network site, as well as the academic center for patients with neurodegenerative diseases. Neurologists nationwide consult the EMC regarding patients with RPD or atypical dementia to coordinate diagnostic evaluation for suspected rare causes, including AE and CJD.

Patients were recruited between December 2019 and December 2024 using 2 complementary approaches. First, patients referred to the EMC by neurologists, geriatricians, or general practitioners for suspected RPD were evaluated by the authors (R.W.v.S., M.J.T., H.S., J.d.V., E.G.P.D., F.J.d.J.). In addition, some patients were referred to and seen at Amsterdam University Medical Center (location VUmc), another large academic memory clinic within the Netherlands (R.W.v.S., Y.A.L.P.). Second, patients who were assessed by a neurologist at another Dutch hospital (both general and academic) and suspected of having RPD were discussed with a neurologist from the EMC as part of the national consultation system, at the discretion of the assessing neurologist at the referring hospital. All referrals and national consultations for suspected RPD during the study period were prospectively and consecutively screened for eligibility by a neurologist from the research team (R.W.v.S., M.J.T., H.S.) through a standardized review of medical records and neuroimaging studies.

We selected adult patients who developed dementia within 1 year of symptom onset,^3^ with dementia defined according to 2011 NINCDS-ADRDA criteria.^22^ Patients with clear structural intracranial lesions (e.g., subdural hematoma or space-occupying mass) or typical presentations of psychiatric disorders or delirium with evident causes (e.g., infection) were excluded. Patients with cognitive dysfunction causing substantial interference with daily activities within 1 year were enrolled after informed consent was obtained.

### Clinical and Imaging Data

Once enrolled, information was prospectively collected concerning demographic characteristics (i.e., age at presentation and sex), medical history, clinical presentation, level of functioning (modified Rankin Scale [mRS]^23^), ancillary testing, and treatment strategies from prospective review of medical records. Clinical symptoms were assessed at first presentation (i.e., presenting phenotype) and throughout the course of clinical evaluation. Next to the prominent dementia symptoms, clinical presentations were categorized into 5 different subphenotypes, according to the most dominant supportive symptom: seizures; movement disorders; psychiatric; pure cognitive; or cerebellar/brainstem (i.e., infratentorial) localization. Faciobrachial dystonic seizures (FBDSs) were classified as seizures (not movement disorders).^24^ Cerebellar ataxia was assessed separately from other movement disorders because of differing etiology.

Neuroimaging features were assessed through direct examination of the MRI scans. Mesiotemporal T2/fluid-attenuated inversion recovery (FLAIR) hyperintensities on brain MRI were considered suggestive of AE after excluding additional atypical features (i.e., enhancement, restricted diffusion) or MRI abnormalities typical for a specific AE subtype (i.e., perivascular enhancement as described in autoimmune glial fibrillary acidic protein (GFAP) astrocytopathy or multifocal corticosubcortical T2/FLAIR hyperintensities as seen in anti–GABAA-R encephalitis).^18,25-28^ In patients who underwent brain biopsy as part of the evaluation, neuropathologic reports were reviewed and discussed with a neuropathologist and the findings were integrated with other ancillary tests, as previously described.^29^ Clinical diagnoses were validated through autopsy, when performed.

### Laboratory Studies

All patients underwent extensive neuronal and glial autoantibody testing in serum and CSF using cell-based assays (CBAs) and immunohistochemistry (IHC).^30,31^ Cell surface autoantibodies were tested using commercial CBAs (Euroimmun, Lübeck, Germany) at the ISO 15189–accredited diagnostic laboratory at EMC or in-house CBAs. Samples with positive CBAs were considered positive when confirmed by IHC.^30,31^ Anti-GFAP–positive CSF samples were confirmed using a cerebellum (primate) slide (Inova Diagnostics, San Diego, CA). CSF total tau (t-tau), phosphorylated tau-181 (p-tau), 14-3-3, and real-time quaking-induced conversion (RT-QuIC) on prion protein were performed on request by the treating physician in the ISO 15189–accredited laboratory at the Radboud UMC. t-tau and p-tau were quantified using ELISAs (Lumipulse Fujibero, Ghent, Belgium). Abnormality was defined by cutoffs: t-tau >400 pg/mL, p-tau >64 pg/mL, and t-tau/p-tau ratio >30. t-tau levels above the level of quantification (i.e., >1,300 pg/mL) were considered to be extremely elevated.

### Definitions

Medical records and neuroimaging were reviewed by 3 neurologists specialized in neurodegenerative diseases or neuroinflammatory disorders and final diagnoses established through consensus (R.W.v.S., H.S., M.J.T.). Patients with positive glial/neuronal autoantibodies and a compatible clinical syndrome were classified as AE-RPD. The 2016 clinical AE criteria were applied for probable seronegative AE (SN-AE).^18^ Established criteria were used to define other neuroinflammatory disorders,^18,32^ neurodegenerative diseases,^22,33-36^ CJD,^37^ and psychiatric disorders.^38^ Diagnoses were categorized as potential treatment-responsive or nonresponsive. Treatment responsiveness was defined as evidence from the published literature that disease-specific treatments (e.g., immunotherapy and antibiotics) have the potential to result in sustained and clinically meaningful improvement in cognitive function, with potential recovery toward functional independence.^1^ Autoimmune/inflammatory conditions, metabolic/toxic disturbances, primary psychiatric disorders, CNS lymphomas, CNS infections, epilepsy, and dural arteriovenous fistula were classified as treatment-responsive. Neurodegenerative diseases, vascular cognitive impairment and dementia, and World Health Organization grade 4 glioma were categorized as nonresponsive.^1^ In a few patients without known diagnoses (n = 7), treatment responsiveness was based on (sustained) effectiveness of previous treatments (n = 1).

### Statistical Analysis

Statistical analyses were performed with IBM SPSS 25.0 (SPSS Inc., Chicago, IL) and R statistical software (version 4.3.2; R Core Team, Vienna, Austria, 2023). The Pearson χ^2^ test or the Fisher-Freeman-Halton test, when appropriate, was used for patient characteristic analysis and group comparisons, encompassing categorical data. *p* Values were 2-sided and considered statistically significant when below 0.05.

### Standard Protocol Approvals, Registrations, and Patient Consents

Written informed consent for usage of medical information for research purposes was obtained from participants or their caregivers. The study protocol was approved by the institutional review board (NL58014.078.18) and performed according to the Strengthening the Reporting of Observational Studies in Epidemiology reporting guideline for observational research.^39^

### Data Availability

Anonymized study data will be shared pending review of a request to the corresponding author from qualified individuals.

## Results

### Patient Characteristics and Diagnostic Categories

**Figure 1:**
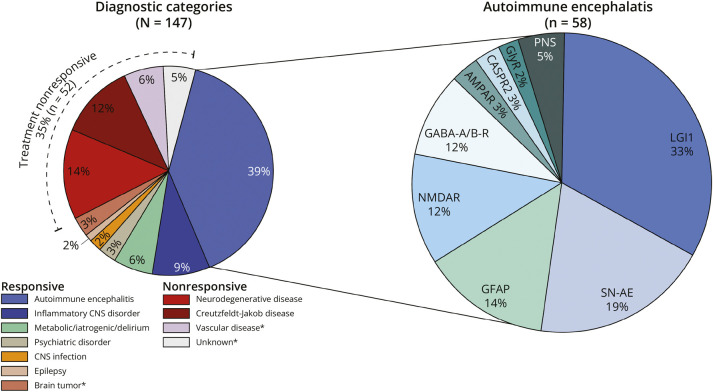
Pie Charts Showing Different Diagnostic Categories (All Diagnoses and Autoimmune Encephalitis) AMPAR = AMPA receptor; CASPR2 = contactin-associated protein-like 2; GFAP = glial fibrillary acidic protein; GlyR = glycine receptor; LGI1 = leucine-rich, glioma-inactivated 1; NMDAR = NMDA receptor; PNS = paraneoplastic neurologic syndrome; SN-AE = seronegative autoimmune encephalitis. *The categories included both treatment-responsive and nonresponsive diagnoses. Specific diagnoses per diagnostic subcategory are provided in eTable 1.

A total of 286 patients with suspected RPD were screened for eligibility, of whom 147 (51%) met criteria for RPD and were included. Ninety of 147 patients (61%) were evaluated and enrolled at the EMC or Amsterdam University Medical Center (location VUmc), or were seen by the authors. Sixty-seven patients (46%) were female (Table 1). The median age was 67 years (interquartile range [IQR] 62–75 years, range 36–86). Time from symptom onset to first presentation was 7 weeks (IQR 1–8 weeks, range 0–62); the median follow-up was 33 months (IQR 19–44 months, range 0.5–62). Paired serum and CSF were tested for neuronal/glial autoantibodies in 146 of 147 patients (99%); only serum was tested in the remaining patient. Ninety-five of 147 patients (65%) were diagnosed with a potentially treatment-responsive cause of RPD (Figure 1, Table 2). AE was the most common treatment-responsive cause of RPD (58/95; 61%), followed by other neuroinflammatory disorders (13/95; 14%) and metabolic disturbances, intoxications, or delirium (combined 9/95; 10%). Neurodegenerative diseases (20/52; 38%) and CJD (17/52; 33%) represented the most common nonresponsive causes of RPD. Autopsy was performed in 8 of 147 patients (5%), showing 100% concordance between clinical and neuropathologic diagnoses (CJD 5/8; 62%, neurodegenerative disease 2/8; 25%, AE 1/8; 13%).

**Table 1:** Patient Characteristics at First Presentation With Rapidly Progressive Dementia: AE vs Other Diagnoses

	All (N = 147)	AE (N = 58; 39%)	Other (N = 89; 61%)	*p* Value
Female sex, n (%)	67 (46)	26 (45)	41 (46)	0.88
Age at presentation, y, median IQR; range	67; 62–75; 36–87	68; 62–74; 45–87	67; 62–73; 36–87	0.48
Medical history, n (%)				
Autoimmune disease	41 (28)	15 (26)	29 (29)	0.66
Systemic malignancy	32 (22)	11 (19)	21 (24)	0.51
Initial presentation, n (%)				
Prodromal symptoms	43 (29)	18 (31)	25 (28)	0.70
Behavioral symptoms	66 (45)	21 (36)	45 (51)	0.08
Psychiatric symptoms	53 (36)	24 (41)	29 (33)	0.28
Hallucinations	30 (20)	15 (26)	15 (17)	0.19
Delusions	19 (13)	10 (17)	9 (10)	0.21
Agitation	21 (14)	11 (19)	10 (11)	0.19
Mania	3 (2)	1 (2)	2 (2)	0.83
Sleeping disorders	36 (25)	14 (24)	22 (25)	0.94
Movement disorders	32 (22)	10 (17)	22 (25)	0.28
Myoclonus	12 (8)	4 (7)	8 (9)	0.65
Parkinsonism	9 (6)	2 (2)	7 (8)	0.23
New-onset seizures	29 (20)	20 (34)	9 (10)	<0.001^c^
Focal^a^	17 (12)	13 (22)	4 (5)	<0.001^c^
Generalized	17 (12)	11 (19)	6 (6)	0.024^c^
Cerebellar/brainstem symptoms	24 (16)	5 (9)	19 (21)	0.041^c^
Autonomous symptoms	10 (7)	4 (7)	6 (7)	0.61
Impaired consciousness	10 (7)	6 (10)	4 (5)	0.15
Hyponatremia (<136 mmol/L)	36 (25)	21 (36)	15 (17)	0.008^c^
MRI brain performed, n (%)	146 (99)	57 (98)	89 (100)	0.21
Mesiotemporal hyperintensities^b^	37 (25)	30 (53)	7 (8)	<0.001^c^
Unilateral	11 (8)	7 (12)	4 (5)	0.082
Bilateral	26 (18)	23 (40)	3 (3)	<0.001^c^
Enhancement	13/71 (18)	6/30 (20)	7/41 (17)	0.75
Restricted diffusion	18 (12)	2 (4)	16 (18)	0.009^c^
≥2 cortical regions	8 (6)	0 (0)	7 (8)	0.028^c^
Caudate/putamen	7 (5)	0 (0)	7 (8)	0.028^c^
CSF performed, n (%)	146 (99)	58 (100)	88 (99)	0.42
WBC >5/μL	61 (42)	33 (58)	28 (32)	0.002^c^
CSF-specific oligoclonal bands	24 (38)	12/23 (52)	12/40 (30)	0.081
EEG performed, n (%)	101 (69)	39 (67)	62 (71)	0.76
Epileptic abnormalities	16 (16)	10 (26)	6 (10)	0.025^c^
PSWCs	4 (4)	0 (0)	4 (6)	0.15
Systemic tumor, n (%)	15 (10)	9 (16)	6 (7)	0.076
mRS score, median; IQR; range	3; 3–4; 2–5	3; 3–3; 2–5	3; 3–4; 2–5	0.39
Duration between onset and dementia, median; IQR; range	13; 3–25; 0–54	13; 4–24; 0–54	13; 3–26; 0–50	0.64
Dementia <3 mo, n (%)	69 (47)	27 (47)	42 (47)	0.94

Abbreviations: FLAIR = fluid-attenuated inversion recovery; IQR = interquartile range; mRS = modified Rankin Scale; PSWCs = periodic sharp-wave complexes; WBC = white blood cell count.

a Three patients with faciobrachial dystonic seizure.

b T2/FLAIR. eTable 2 lists the comparison between treatment-responsive and nonresponsive diagnoses. eTable 3 lists the comparison between AE and Creutzfeldt-Jakob disease.

c *p* Values showing significance (*p* < 0.05) between AE patients and other.

**Table 2:** Specific Diagnoses per Diagnostic Subcategory

Specific diagnoses (n = 147)	
Treatment-responsive (n = 95; 65%)	Nonresponsive (n = 52; 35%)
Autoimmune encephalitis^a^ 58 (61%) LGI1 19 (33%) SN-AE 11 (19%) GFAP 8 (14%) NMDAR 7 (12%) GABA_B_-R 4 (7%) PNS^b^ 3 (5%) CASPR2 2 (3%) AMPAR 2 (3%) GABA_A_-R 1 (2%) GlyR 1 (2%)	Neurodegenerative disease 20 (39%) Alzheimer disease 7 (35%) Dementia with Lewy bodies 6 (30%) Frontotemporal dementia 4 (20%) Progressive supranuclear palsy 3 (15%)
Inflammatory CNS disorder 13 (14%) Probable neuroinflammatory disorder 9 (69%) Hashimoto encephalopathy 2 (15%) PACNS 1 (8%) Anti–TNF-induced demyelination 1 (8%)	Creutzfeldt-Jakob disease 17 (33%)
Metabolic/toxic/delirium 9 (10%) Delirium with underlying systemic infection 3 (33%) Hyponatremia 2 (22%) Drug-induced cognitive impairment 3 (33%) Hyperthyroidism 1 (11%)	
Primary psychiatric disorder 4 (4%) Depressive disorder 2 (50%) Psychotic disorder 1 (25%) Anxiety disorder 1 (25%)	
Brain tumor 4 (4%) PCNSL 4 (100%)	Brain tumor 1 (2%) Glioma WHO grade 4 1 (100%)
CNS infection 3 (3%) Neurosyphilis 2 (67%) Neuroborreliosis 1 (33%)	
Epilepsy with a noninflammatory cause 2 (2%)	
Vascular 1 (1%)	Vascular 8 (15%)
Dural arteriovenous fistula 1 (100%)	Vascular dementia 8 (100%)
Unknown^c^ 1 (1%)	Unknown^d^ 6 (12%)

Abbreviations: AMPAR = AMPA receptor; Anti-TNF = anti–tumor necrosis factor; CASPR2 = contactin-associated protein-like 2; GABA_A_-R = GABA_A_-receptor; GABA_B_-R = GABA_B_-receptor; GFAP = glial fibrillary acidic protein; GlyR = glycine receptor; LGI1 = leucine-rich, glioma-inactivated 1; NMDAR = NMDA receptor; PACNS = primary angiitis of the CNS; PCNSL = primary CNS lymphoma; SN-AE = seronegative autoimmune encephalitis; WHO = World Health Organization.

a Autoantibody studies included testing for anti-IgLON5 antibodies by cell-based assays and immunohistochemistry.

b Ma2 (1), KLHL11 (1), seronegative PNS (1).

c Patient with a high suspicion of PCNSL with enhancement on brain MRI and mild pleocytosis deceased before brain biopsy.

d All patients with a high suspicion of a neurodegenerative disease with atrophy on brain MRI and absence of signs indicative of a treatment-responsive disorder.

### Clinical Presentation and Ancillary Testing

**Figure 2:**
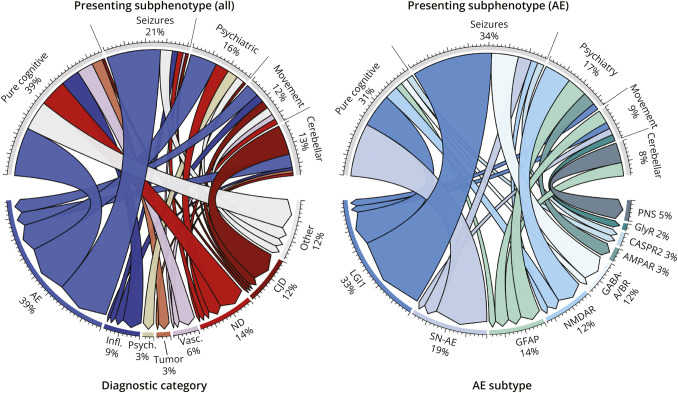
Chord Diagram Showing Relationship Between Presenting Subphenotypes and Underlying Diagnoses (All Diagnoses and AE) AE = autoimmune encephalitis; AMPAR = AMPA receptor; CASPR2 = contactin-associated protein-like 2; CJD = Creutzfeldt-Jakob disease; GFAP = glial fibrillary acidic protein; GlyR = glycine receptor; Infl. = inflammatory CNS disorder; LGI1 = leucine-rich, glioma-inactivated 1; ND = neurodegenerative disease; NMDAR = NMDA receptor; presenting phenotype = symptoms at first presentation; Psych. = psychiatric disorder; SN-AE = seronegative autoimmune encephalitis; Vasc. = vascular.

Seizures were more common in AE-RPD compared with other diagnoses (34% vs 10%; *p* < 0.001; Table 1), and AE was the most common underlying disorder in patients with RPD and seizures (20/29; 69%; Figure 2). Cerebellar/brainstem signs were more common in non-AE diagnoses (19/89; 21% vs 5/58; 9%; *p* = 0.041), including 11 of 19 patients (58%) with RPD attributed to CJD (Figure 2). Hyponatremia, mesiotemporal T2/FLAIR hyperintensities on MRI, and CSF pleocytosis were more common in patients with AE-RPD vs other diagnoses (Table 1). By contrast, restricted diffusion on brain MRI was more frequent in non-AE diagnoses (18% vs 4%; *p* = 0.009), particularly in ≥2 cortical regions or caudate/putamen (8% vs 0%; *p* = 0.028)—findings exclusively seen in patients with CJD (7/7; 100%).

**Figure 3:**
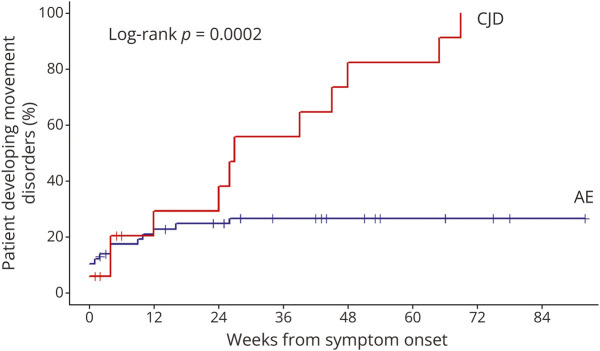
Time Evolution of Movement Disorders in AE and Creutzfeldt-Jakob Disease AE = autoimmune encephalitis; CJD = Creutzfeldt-Jakob disease. Last follow-up or death without the development of movement disorder was treated as censoring events and is indicated by plus signs (+).

The median time from symptom onset to dementia was shorter in patients with potentially treatment-responsive RPD than in those with nonresponsive RPD (8 vs 22 weeks), corresponding to a median difference of 14 weeks (95% CI 5.5–19.0; *p* < 0.001). Most patients with treatment-responsive causes met RPD criteria within 3 months of symptomatic onset (58% vs 27%; *p* < 0.001; eTable 1). Abnormal movements were identified at first presentation in similar proportions of patients with AE-RPD (17%) and CJD (35%; *p* = 0.11; eTable 2) but were more frequent over the course of RPD in patients with CJD (71%) vs other causes (26%; *p* < 0.001), with a higher hazard of developing abnormal movements in those with CJD compared with AE (hazard ratio 3.9, 95% CI 1.8–8.5; Figure 3). Most patients with AE-RPD (80%) developed movement disorders within 3 months after symptom onset, whereas patients with CJD-RPD mostly developed these later (75% beyond 3 months; *p* = 0.004; Figure 3). CSF t-tau levels above the level of quantification (>1300 pg/mL) and t-tau/p-tau >30 were found in 7 patients with a diagnosis other than CJD (eTable 3). Five of these patients (63%) were diagnosed with AE, including SN-AE (n = 3), anti-GABA_A_R, and anti-GABA_B_R (each, n = 1). These patients showed signs considered atypical for CJD, including seizures (2/5; 40%), MRI suggestive of AE without restricted diffusion, and CSF pleocytosis (5/5; 100%). RT-QuIC was exclusively positive in patients with CJD but was also negative in 3 patients with definite (i.e., autopsy-proven) sporadic CJD.

### AE Subtypes

**Figure 4:**
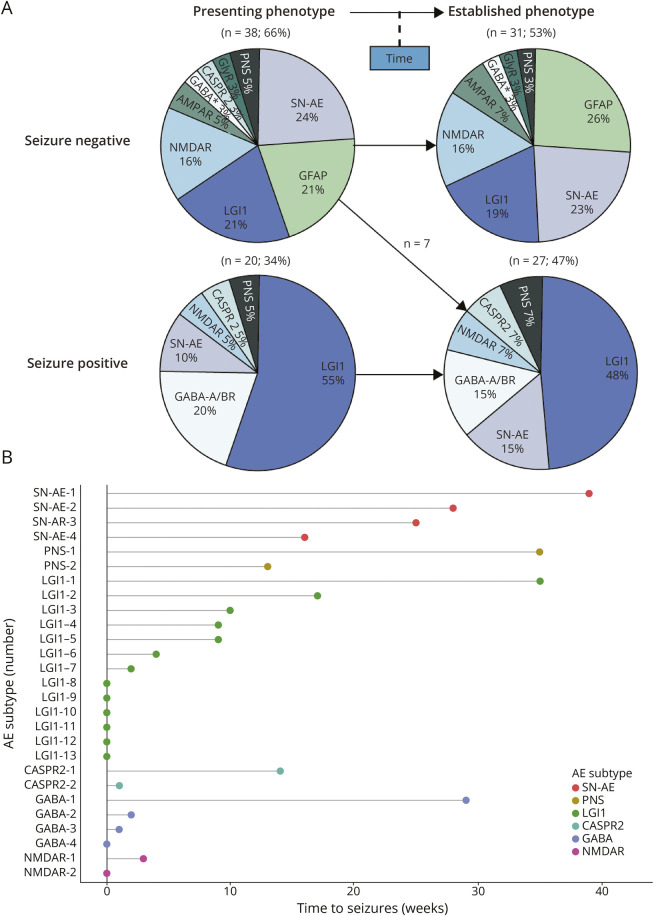
Seizures in Patients With Rapidly Progressive Dementia Due to Autoimmune Encephalitis (A) Autoimmune Encephalitis subtypes in seizure-positive vs seizure-negative patients (at first presentation and during complete period of diagnostic evaluation). (B) Time from symptom onset to seizures in seizure-positive rapidly progressive dementia due to autoimmune encephalitis. Presenting phenotype = symptoms at first presentation. Established phenotype = symptoms during complete period of diagnostic evaluation. AMPAR = AMPA receptor; CASPR2 = contactin-associated protein-like 2; GFAP = glial fibrillary acidic protein; GlyR = glycine receptor; LGI1 = leucine-rich, glioma-inactivated 1; NMDAR = NMDA receptor; PNS = paraneoplastic neurologic syndrome; SN-AE = seronegative autoimmune encephalitis. *Anti-GABA_A/B_ receptor encephalitis.

Anti–leucine-rich, glioma-inactivated 1 (LGI1) (19/58; 33%), SN-AE (11/58; 19%), and autoimmune GFAP astrocytopathy (8/58; 14%) were the 3 most common subtypes in AE-RPD (Figure 1, Table 2). The seizure subphenotype represented the most common clinical presentation of AE-RPD (20/58; 34%, Figure 2). Among these, anti-LGI1 (11/20; 55%) was the most common underlying subtype, followed by anti–GABA_A/B_-R (4/20; 20%; Figure 2). At first presentation, 13 of 20 patients with AE-RPD and seizures (65%) had focal seizures, including 9 (69%) without generalized seizures (eFigure 1). Six of 13 patients (46%) presented with subtle nonmotor, autonomic, or dyscognitive seizures, and 3 of 13 patients (23%) presented with FBDS. Seizures were missed at first presentation in 5 of 20 patients with AE (25%). Anti-LGI1 was the most common underlying AE subtype in patients with focal seizures (9/13; 69%). Ultimately, 27 of 58 patients with AE-RPD (47%) developed seizures over the illness course (Figure 4A), with 17 of 27 (63%) experiencing seizures within 3 months of symptom onset (median 4 weeks; IQR 0–17 weeks, range 0–39; Figure 4B). Eleven of 13 seizure-positive anti-LGI1 patients (85%) developed seizures within 3 months.

In the pure cognitive subphenotype and among all seizure-negative patients, SN-AE was the most common AE-RPD subtype (8/18; 44% and 7/31; 23%, respectively; Figures 2 and 4A). Eight of 11 patients with SN-AE (73%) presented with a pure cognitive subphenotype, while additional neurologic symptoms were present in the remaining patients, including seizures (2/11; 18%) or movement disorders (1/11; 9%). Ultimately, 4 of 12 patients with SN-AE (33%) developed seizures, all occurring more than 3 months after symptom onset (Figure 4B).

### Autoimmune GFAP Astrocytopathy

Autoimmune GFAP astrocytopathy was the most common subtype in seizure-negative AE-RPD patients (8/31; 26%, Figure 4A, Table 3). Six of 8 patients with autoimmune GFAP astrocytopathy (75%) were male, with a median age at onset of 66 years (IQR 60–72, range 56–83). Prodromal weight loss was reported by 4 of 8 patients (50%). Seven of 8 patients (88%) had clear neurologic or psychiatric signs, in addition to RPD. Psychiatric symptoms were present in 4 of 8 patients (50%), mainly delusions and visual hallucinations dominating the phenotype in 3 of 8 patients (38%). Myoclonus (3/8; 38%) and tremor (2/8; 25%) were the most common movement disorders. Brain MRI was abnormal in 6 of 8 patients (75%), showing perivascular enhancement (2/8; 25%), mesiotemporal hyperintensities (5/8; 63%), and pachymeningeal enhancement (1/8; 13%). CSF pleocytosis was observed in all patients. A good outcome (mRS score ≤2 at 1 year after immunotherapy) was observed in 6 of 8 patients (75%), including 1 patient who was treated with immunotherapies for more than 1 year after symptom onset.

**Table 3:** Characteristics of Patients With Rapidly Progressive Dementia Due to Autoimmune GFAP astrocytipathy

Patient no.	Sex; age (range)	Subphenotype	Clinical presentation	mRS score at nadir	Time to dementia (wk)	Ancillary testing	Tumor	ITX	Time to treatment (wk)	mRS 1 y after ITX
1	M; 50–60	Psychiatric	Cognitive decline, visual hallucinations, delusions, tremor, weight loss	3	43	MRI + C: perivascular enhancement, bilateral mesiotemporal hyperintensities CSF: L 16/mm^3^, OCB+	No	IVMP, IVIG, oral prednisone, rituximab	67	1
2	V; 60–70	Psychiatric	Cognitive decline, visual hallucinations, depressive symptoms	3	30	MRI + C: bilateral mesiotemporal hyperintensities CSF: L 35/mm^3^, OCB NA	No	IVMP, IVIG, oral prednisone, rituximab	31	3
3	M; 70–80	Psychiatric	Cognitive decline, delusions, obsessive behavior, weight loss, gait disorder	3	34	MRI + C: generalized atrophy CSF: L 74/mm^3^, OCB+	No	IVMP, IVIG, oral prednisone	35	3
4	M; 60–70	Cerebellar ataxia	Cognitive decline, weight loss, cerebellar ataxia, visual hallucinations, myoclonus	4	8	MRI + C: bilateral mesiotemporal hyperintensities CSF: L 151/mm^3^, OCB NA	No	IVMP, IVIG, oral prednisone	9	1
5	M; 60–70	Cerebellar ataxia	Cognitive decline, cerebellar ataxia, weight loss	3	14	MRI + C: bilateral mesiotemporal and cortical hyperintensities CSF: 41, OCB NA	No	IVMP, IVIG, oral prednisone	22	1
6	V; 60–70	Movement disorder	Cognitive decline, tremor	3	19	MRI + C: perivascular enhancement, unilateral mesiotemporal hyperintensity CSF: L 49/mm^3^, OCB+	No	IVMP, IVIG, oral prednisone, MMF, AZA	23	1
7	M; 40–50	Movement disorder	Cognitive decline, myoclonus, fever, impaired consciousness	5	1	MRI + C: pachymeningeal enhancement CSF: L 593/mm^3^, OCB−	No	IVIG, oral prednisone	2	2
8	M; 80–90	Pure cognitive	Cognitive decline	3	3	MRI + C: small vessel disease CSF: L 207/mm^3^, OCB−	No	IVMP, IVIG, oral prednisone	1	2

Abbreviations: AZA = azathioprine; GFAP = glial fibrillary acidic protein; ITX = immunotherapy; IVIG = IV immunoglobulin; IVMP = IV methylprednisone; L = leukocytes; MMF = mycophenolate mofetil; MRI + C = MRI with contrast; mRS = modified Rankin Scale; NA = not applicable; OCB = unique oligoclonal band.

## Discussion

In this prospective multicenter cohort study, we determined the frequency of AE in patients presenting with RPD and comprehensively characterized the phenotypes of AE-RPD, compared with other causes of RPD. AE was the most common treatment-responsive cause of RPD, accounting for approximately 40% of diagnoses, being higher than previously reported.^1,6,7,9,21^ Among patients with AE-RPD, anti-LGI1, SN-AE, and autoimmune GFAP astrocytopathy were the 3 most common AE subtypes, altogether representing approximately 65% of patients with AE-RPD. We applied an extensive autoantibody testing methodology, as paired serum and CSF were tested in 99% of patients using multiple techniques, resulting in optimal autoantibody detection.^30,31^ Our findings emphasize that AE should be considered in patients presenting with RPD; targeted ancillary testing should be performed dependent on specific patient characteristics.

The most apparent early clinical difference between AE and other causes of RPD was the high occurrence of seizures (34%) in patients with AE-RPD.^15,24,26^ Anti-LGI1 accounted for almost one-third of AE-RPD cases and almost one-half of cases in patients with seizures at presentation. This observation emphasizes the importance of recognizing seizures in patients with RPD.^9,26^ Seizures are also recognized in patients with RPD attributed to neurodegenerative diseases (∼10%) but usually occurred later in the illness course.^40^ Although seizures are usually easily recognized, we identified 2 important diagnostic pitfalls. First, around half of patients presented solely with focal seizures. Focal seizures may be easily missed or misdiagnosed because of the lower frequency of motor signs (absent in a substantial proportion of patients) and possible manifestation with subtle signs, such as piloerection.^41^ In particular, frequent (>5 per day), stereotyped episodes should raise suspicion for anti-LGI1 encephalitis and prompt autoantibody testing, which can be reliably performed in serum because of its high diagnostic accuracy.^24,26^ Second, approximately 25% of patients only developed seizures after their initial presentation and around 20% even more than 6 months after onset of symptoms. We recommend proactive clinical monitoring for potential seizures in patients with RPD, even if absent during the first evaluation.

Early identification of patients with AE-RPD is particularly challenging if seizures are absent because other symptoms (e.g., psychosis and focal deficits) are usually less specific.

Autoimmune GFAP astrocytopathy was the most common subtype in seizure-negative AE-RPD patients, of whom most (∼90%) had striking additional (i.e., noncognitive) signs. The psychiatric phenotype was the most common presentation of autoimmune GFAP astrocytopathy, representing a clearly defined clinical syndrome characterized by severe psychotic symptoms and rapid cognitive decline. In previous descriptions of autoimmune GFAP astrocytopathy, cognitive and psychiatric symptoms were reported in 10%–35% of patients.^27,42^ In RPD accompanied by movement disorders, autoimmune GFAP astrocytopathy was the most common subtype, with myoclonus and tremor as most common movement disorders. We observed that movement disorders are typically an early symptom of AE (i.e., within 3 months), whereas in CJD, these symptoms generally appear at later stages.^43^ MRI and CSF findings were mostly abnormal in autoimmune GFAP astrocytopathy, facilitating early detection. A striking radiologic feature in patients with autoimmune GFAP astrocytopathy in this study was the high occurrence (∼60%) of mesiotemporal T2/FLAIR hyperintensities on brain MRI. Although the involvement of the temporal pole has been reported in some patients with autoimmune GFAP astrocytopathy,^42^ a distinct radiologic pattern of limbic encephalitis has not been described earlier, highlighting the expanding clinicoradiologic profile of this AE-RPD subtype. In accordance with previous research, particularly the presence of a CSF pleocytosis was highly sensitive.^27,42^ Identification of autoimmune GFAP astrocytopathy in RPD is crucial because immunotherapy resulted in favorable outcomes in 75% of patients, making this AE subtype a highly treatment-responsive cause of RPD. It is important to note that GFAP antibodies are not included within all commercial test panels. Therefore, it is strongly recommended to test specifically for GFAP antibodies in seizure-negative RPD patients, especially when accompanied by psychotic symptoms, early-onset movement disorders, or cerebellar ataxia in combination with CSF pleocytosis. GFAP antibodies should be tested in CSF and confirmed by tissue analysis, in view of limited sensitivity and specificity in serum.^27^

SN-AE was a relatively common cause of RPD in our series, with 11 of 147 patients of RPD (7%) meeting the 2016 clinical criteria,^18,26^ which maintains high specificity when applied rigorously.^26^ Early identification of patients with RPD attributed to SN-AE is challenging, given that approximately 75% of patients presented with isolated cognitive symptoms and specific biomarkers are not available. Seizures were relatively rare and occurred at a later stage (i.e., after 6 months), consistent with earlier reports.^44^ However, higher frequencies of seizures (∼80%) are also reported in SN-AE,^15^ possibly explained by differences in study selection criteria. Seizure-negative SN-AE might be more prone to diagnostic delays and subsequent development of severe cognitive decline. This could help explain the relatively high number of patients with SN-AE in our study because these patients were possibly more frequently suspected of having RPD and referred to memory clinics. In 2023, a large cohort study showed that the outcome of SN-AE was worse compared with anti-NMDAR encephalitis and delay of immunotherapy (≥1 month) was associated with an unfavorable outcome,^17^ emphasizing the importance of early identification. We demonstrate that SN-AE is an important differential diagnostic consideration in patients with RPD—especially patients with isolated cognitive symptoms. However, it is important to emphasize that SN-AE represents a heterogeneous group of diseases, with significant variation in clinical phenotypes and likely diverse underlying pathogenic mechanisms.^45^ Further research is needed to identify specific biomarkers to improve early identification of SN-AE and subcategorize within this heterogeneous group of diseases.^45^

Several patients with treatment-responsive causes of RPD, mostly AE, showed markedly elevated CSF t-tau levels and increased t-tau/p-tau ratios—findings that are generally considered specific for CJD.^15,46^ It is important to note that all these patients exhibited clinical or radiologic signs that were atypical for CJD. Tau levels most likely reflect the severity of synaptic dysfunction in these patients, rather than a specific etiology.^47^ These findings underscore the need for accurate diagnostic tests and emphasize the essential role of critically interpreting test results in relation to the clinical presentation.

Neuronal antibodies studies in this study included testing for anti-IgLON5 autoantibodies, as cognitive decline can be the presenting symptom of this AE subtype.^48^ Notably, no patients with anti-IgLON5 disease were identified in our study, which is probably explained by the fact that the cognitive symptoms in anti-IgLON5 disease generally follow a more insidious course,^13^ complicating early diagnosis in dementia patients. In patients with a slowly progressive course of cognitive decline, testing for anti-IgLON5 autoantibodies should specifically be considered if additional signs are present, in particular movement disorders, sleep disorder, or bulbar symptoms.^13,48^

The EMC is the national referral center for neuronal autoantibody testing and AE in the Netherlands, which likely contributed to the high proportion of patients with AE-RPD in our cohort vs others.^6,21^ However, comparable frequencies (∼30%) of AE-RPD have been described previously in cohorts from the United States and Brazil.^1,7,9^ Collectively, these findings affirm the relevancy of this group of diseases in patients undergoing evaluation for RPD. Selection bias toward atypical CJD cases probably occurred because typical CJD cases might have never been referred nor discussed with us. The lower sensitivity of the RT-QuIC in our study compared with previous research may reflect this bias.^37,43^ Therefore, our findings should be interpreted with caution because they may not be directly extrapolated to other settings (e.g., specialized prion centers and lower and middle-income countries), which strongly influences the prevalence of RPD causes. This is illustrated by previous research: neuroinfections were the leading cause of RPD in a large Indian cohort (39%),^49^ whereas neurodegenerative diseases and CJD were more frequent in another RPD cohort.^6^ However, in this multicenter study, patients were enrolled in many different settings, both specialized and nonspecialized, contributing to the heterogeneity of the cohort.

Specific paraclinical features that could aid in diagnosing neurodegenerative diseases were not included because our focus was on AE-RPD. For example, substantial brain atrophy on MRI has been linked to ongoing decline in suspected RPD cases,^9^ whereas CSF biomarkers other than t-tau and p-tau (e.g., amyloid-β 42/40 ratio, GFAP, and neurofilament light) may provide valuable diagnostic insight into patients with RPD.^50^ Furthermore, neuropsychological assessments were not included in this study because they were only conducted in a minority of patients, primarily due to the rapidity of decline and severity of cognitive deficits, which limits interpretation of these tests in patients with RPD. Further exploration and comparison of profiles of cognitive dysfunction across different causes of RPD would be valuable for future research.

In summary, AE is the most common treatment-responsive cause of RPD and anti-LGI1 encephalitis is the most common subtype, in particular in patients with RPD and early seizures. Comprehensive testing for disease-associated autoantibodies is strongly recommended in all patients with RPD and should include testing for anti-GFAP antibodies in CSF.

## Glossary

AE: autoimmune encephalitis
AE-RPD: AE subtypes in patients with RPD
CBA: cell-based assay
CJD: Creutzfeldt-Jakob disease
EMC: Erasmus University Medical Center
FBDS: faciobrachial dystonic seizure
FLAIR: fluid-attenuated inversion recovery
GFAP: glial fibrillary acidic protein
IHC: immunohistochemistry
IQR: interquartile range
LGI1: leucine-rich, glioma-inactivated 1
mRS: modified Rankin Scale
p-tau: phosphorylated tau-181
RPD: rapidly progressive dementia
RT-QuIC: real-time quaking-induced conversion
SN-AE: seronegative AE
t-tau: total tau
